# Effects of in vitro‐induced drug resistance on the virulence of *Streptococcus*

**DOI:** 10.1002/vms3.404

**Published:** 2020-12-13

**Authors:** Yue‐Xia Ding, Qun Wu, Yan Guo, Man Li, Pei‐Feng Li, Yi Ma, Wen‐Chao Liu

**Affiliations:** ^1^ College of Coastal Agricultural Sciences Guangdong Ocean University Zhanjiang PR China; ^2^ Department of Veterinary Pharmacology & Toxicology College of Veterinary Medicine Inner Mongolia Agricultural University Hohhot PR China; ^3^ Laboratory of Clinical Diagnosis and Treatment Techniques for Animal Disease Ministry of Agriculture Hohhot PR China; ^4^ Research Institute of Agricultural Machinery Chinese Academy of Tropical Agricultural Sciences Zhanjiang PR China; ^5^ Maoming Branch Guangdong Laboratory for Lingnan Modern Agriculture Maoming PR China

**Keywords:** drug resistance, *Streptococcus*, virulence

## Abstract

This study aimed to evaluate the effects of in vitro‐induced drug resistance on the virulence of *Streptococcus*. Micro‐dilution method was used to determine the minimal inhibitory concentration (MIC). In vitro‐induced drug resistance was conducted for *S. agalactiae* (CVCC1886) and *S. dysgalactiae* (CVCC3701) by gradually increasing the antimicrobial concentration (strains were from IVDC, China). PCR was used to detect the resistance and virulence genes of the strains before and after resistance induction. Colony morphology was observed to compare the physiological and biochemical properties of the strains. A total of 88 clean‐grade Kunming mice (obtained from Inner Mongolia University, Hohhot, China) were used in half of the lethal dose (LD50) test for detecting the changes in virulence of strains. The results showed that *S. agalactiae* (CVCC1886) and *S. dysgalactiae* (CVCC3701) developed resistance against seven kinds of antibiotics, respectively. Resistance and virulence genes of CVCC3701 were changed when treated by the Penicillin‐inducing. The growth of the CVCC3701‐PEN was decreased compared to the CVCC3701. Virulence test in mice indicated that the LD50 of CVCC3701 before induction and CVCC3701‐PEN after induction were 5.45 × 10^6^ and 5.82 × 10^8^ CFU/ml, respectively. Compared with the untreated bacteria, the bacterial virulence was reduced 1.1 × 10^2^ times after resistance induction. In conclusion, *S. dysgalactiae* (CVCC3701) is a susceptible strain of drug resistance to antibiotics, in vitro‐induced drug resistance reduced the virulence of CVCC3701, but the virulence is still existing and also could result in the death of mice. For public health safety, it must be alert to the emergence of drug resistance of *Streptococcus* in animal production.

## Graphical abstract

Streptococcus dysgalactiae (CVCC3701) is a susceptible strain of drug resistance to antibiotics, in vitro‐induced drug resistance reduced the virulence of CVCC3701, but the virulence is still exists and also could result in the death of mice. For the public health safety, it must be alert to the emergence of drug resistance of Streptococcus in animal production.

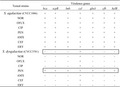

## 1 INTRODUCTION

*Streptococcus agalactiae* (*S. agalactiae*) are the main pathogens that causing mastitis and invasive disease in cows (Jain et al., 2012), whereas *Streptococcus dysgalactiae* (*S. dysgalactiae*) is environmental pathogens (Guérin‐Faublée et al., 2002). Various species of *Streptococcus* are known to be associated with bacterial infections in cattle, pigs, horses, sheep, birds, aquatic mammals, and fishes (Ding et al., 2016). Zoonotic transmission of *S. dysgalactiae* subsp. *Equisimilis* has been previously suggested by Silva et al. (2015), and *S. agalactiae* is also a human pathogen that mainly cause neonatal infections (Poyart et al., 2003). In recent decades, animal production has tended to a high‐density and intensive production model (Guo et al., 2020a; Liu et al., 2020), leading to frequent occurrence of animal diseases, and antibiotics are therefore widely used to prevent and treat animal bacterial infectious diseases (Guo et al., 2020b; Liu et al., 2019). The antibiotic environment in livestock production already existed that was below the antimicrobial concentration and could induce bacterial resistance (Stolker et al., 2013). On the other hand, the sub‐inhibitory concentration of antibiotics will also affect the virulence of bacteria by toxin production, adhesion, movement (Dal Sasso et al., 2002, 2003). In the process of adapting to antibiotics of sub‐inhibitory concentration, ecological characteristics of bacteria will change adaptively, thus showing polymorphism (Friman et al., 2015). In particular, the reproductive performance of cows has been found to decrease as the antimicrobial resistance increases (Guérin‐Faublée et al., 2002). The issue of resistance of *Streptococcus* is not only a great concern in animal health and production, but also exert serious effects on both the environment and public health safety (Tian et al., 2019).

*Streptococcu* possesses a variety of virulence factors that contribute to pathogenicity, such as surface proteins and adhesion factors are involved in adhesion, invasion of host cells, and escape of the immune system (Brooks & Mias, 2018). It has been demonstrated that the surface components of bacteria, including capsulated polysaccharides, protein components (Cα, Cβ, ribose), laminin‐binding proteins (Lmb), and C5a peptidase, which were related to virulence (Maisey et al., 2008). The α‐antigen and β‐antigen were found to be encoded by the *bac* and *bca* genes (Beigverdi et al., 2014; Lindahl et al., 2005). The *cyl* and *cfb* genes encode haemolysin and the cyclic adenosine mono‐phosphate (CAMP) factor, respectively (Dmitriev et al., 2002). However, to the best of our knowledge, the effect of *Streptococcal* resistance on virulence has been poorly described. Therefore, the purpose of this study was to induce drug resistance of *Streptococcus*, and analyse the resistance changes and virulence‐related genes, with the expectation to explore the relationship between drug resistance and virulence, so as to lay a scientific basis for effective therapy of animal diseases caused by *Streptococcal*.

## 2 MATERIALS AND METHODS

### 2.1 Antimicrobial susceptibility test

The *S. agalactiae* (CVCC1886) and *S. dysgalactiae* (CVCC3701) were obtained from the China Institute of Veterinary Drug Control (IVDC, China), and they were revived by subculture on Mueller‐Hinton agar plates supplemented with 5% defibrinated sheep blood and incubated at 37°C for 18–24 hr aerobically in 5% CO_2_ for antimicrobial susceptibility test. Minimal inhibitory concentrations (MICs) were determined using the micro‐dilution method as recommended by the Clinical and Laboratory Standards Institute (CLSI, 2018). This method used Muller Hinton (MH) broth and consisted of manually prepared 96‐well microtitre plates containing the following seven antibiotics from the IVDC: penicillin, amoxicillin, cefalotin, norfloxacin, ofloxacin, ciprofloxacin, erythromycin.

Each antibiotic tested was diluted using a two‐fold dilution pattern and wells containing different concentrations (0.125, 0.25, 0.5, 1.0, 2.0, 4.0, 8.0, 16.0, 32.0, 64.0 and 128 µg/ml) were prepared. The inoculum was prepared by suspending several colonies of *Streptococcus* in MH broth and adjusting the value of optical density at 625 nm (OD625) to 0.1 (about 1 × 10^8^ CFU/ml). The final bacterial concentration was diluted to 1 × 10^6^ CFU/ml of 50 ml per well. The plates were covered and incubated at 37°C for 18–20 hr. At the same time, *S. pneumoniae* ATCC 49,619 was used as the control strain. All susceptibility results were complied with the quality control ranges.

### 2.2 In vitro‐induced drug resistance assay

The in vitro‐induced drug resistance study was based on a previous report (Yao et al., 2018). Briefly, the preserved standard strains were inoculated into the Brain Heart Infusion (BHI) broth with serum, and incubated at 37°C for 6 hr. A small amount of bacterial solution was picked to streak MH agar plates and incubated at 37°C for 16–20 hr. BHI broth containing the sub‐inhibitory concentration of antibiotic was prepared and incubated at 37°C. Meanwhile, the negative control of broth was made and transferred every 3 days. The concentration of the induced antibiotic was gradually increased by two times until the MIC of the test strain rose to the resistance range.

### 2.3 Detection of antimicrobial resistance genes and virulence factors

The resistant strains that displayed phenotypic resistance to antibiotics were further tested for the presence of antimicrobial resistance genes. PCR was used to amplify *GyrA* and *GyrB* genes contributing to quinolone resistance. *Pbp1α*, *Pbp1b* and *Pbp2x* conferring resistance to β‐lactam. *ermA*, *ermB* and *mefA* genes encoding resistance to erythromycin. Primers, amplicon size and annealing temperatures are listed in Table 1. PCR method was also performed to detect virulence genes (*bca, cfb, cyl, glnA, hylB, lmb, scpB*) in strains before and after induction.

**TABLE 1:** Details of the PCR primers used to amplify *Streptococcus* antimicrobial resistance genes and virulence factors

Target genes	Primer sequence (5′–3′)		Amplicon size (bp)	Tm (°C)
	Forward	Reverse		
*GyrA*	AAAACCTGTTCATCGTCGTA	TTCACGTTCACTGCCATC	343	52
*GyrB*	CGGTGTTGTTGTTAATGAT	TCTTGACCTTCTCGCTTGT	198	50
*Pbp1α*	TTGAATTTAGCGATGGAAC	CAGAAGAAGAGTAAGTGCC	489	50
*Pbp1b*	TTTGATGCTAAGGGAGTT	ATACGGTTGATTCGGCTA	268	50
*Pbp2x*	GATGCTACCTGGCTTGAT	CCAGACTTGAGGGCTACA	422	50
*ermA*	TCTAAAAAGCATGTAAAAGAA	CTTCGATAGTTTATTAATATTAGT	645	52
*ermB*	ATTGGAACAGGTAAAGGGC	GAACATCTGTGGTATGGCG	442	50
*mefA*	AGTATCATTAATCACTAGTGC	TTCTTCTGGTACTAAAAGTGG	346	53
*bca*	ACGACTTTCTTCCGTCCACTTAGG	ACGACTTTCTTCCGTCCACTTAGG	535	51
*cfb*	ATGGGATTTGGGATAACTAAGCTAG	AGCGTGTATTCCAGATTTCCTTAT	193	52
*cyl*	ACGGCTTGTCCATAGTAGTGTTTG	AACGACACTGCCATCAGCAC	345	52
*glnA*	ACGTATGAACAGAGTTGGCTATAA	TCCTCTGATAATTGCATTCCAC	471	52
*hylB*	ACAAATGGAACGACGTGACTAT	CACCAATTGGCAGAGCCT	346	52
*lmb*	ACCGTCTGAAATGATGTGG	GATTGACGTTGTCTTCTGC	572	51
*scpB*	CCAAGACTTCAGCCACAAGG	CAATTCCAGCCAATAGCAGC	591	57

### 2.4 Detection of physiological and biochemical properties in strains before and after induction

The conventional microbiological method was used to detect the physiological and biochemical properties, and the growth of bacteria (Cui et al., 2003). The test strains were selected with large differences before and after induction according to the detection of genes, which were streaked on MH agar plates supplemented with 5% defibrinated sheep blood and incubated at 37°C for 18–24 hr to observe colony morphology and hemolysis. Single colony was selected and placed in MH broth that adjusting the bacterial concentration to 1 × 10^8^ CFU/ml. One ml of the tested bacteria solution was added to 99 ml of MH broth with 5% serum for oscillating culture in 37°C. The absorbance value of the solution was measured every 1–14 hr at OD625 nm, and the growth curve was plotted.

### 2.5 Detection of LD50 on tested strains

Half of the lethal dose (LD50) was determined by Bliss method as previous reported (Bliss, 1936). The experimental design was divided into a blank control and two experimental groups, they are shown in Table 2. Briefly, a total of 88 clean‐grade Kunming mice (7 weeks, the body weight is between 18–22 g, purchased from Inner Mongolia University, Hohhot, China) were used in the animal LD50 detection experiment. Firstly, after one week of adaptive feeding, they are divided into equal groups, and half of the male and female in each group. Both drinking water and litter were autoclaved. Each group of five mice, adjust the concentration of bacterial solution, determine LD0 and LD100. Based on the results, expand the number of experimental animals and verify the results. According to the values of LD0 and LD100, three concentrations were set in equal proportions in the middle to form five concentration gradients, with eight mice in each group. After the challenge, observe for 14 days, record the number of deaths, and calculate LD50 according to the Bliss method.

**TABLE 2:** Groups of LD50 test

Tested strains	Groups	Inoculated doses (ml)	Inoculated mode	Numbers
LB Broth	Blank control	0.5	ip	8
*S. dysgalactia* CVCC3701	1‐LD0	0.5	ip	8
	1‐n1LD0	0.5	ip	8
	1‐n12LD0	0.5	ip	8
	1‐n13LD0	0.5	ip	8
	1‐LD100	0.5	ip	8
*S. dysgalactiae* CVCC3701‐ PEN (penicillin)	2‐LD0	0.5	ip	8
	2‐n2LD0	0.5	ip	8
	2‐n22LD0	0.5	ip	8
	2‐n23LD0	0.5	ip	8
	2‐LD100	0.5	ip	8

Abbreviations: ip, intraperitoneal injection; LB Broth, Luria Bertani Broth; LD, lethal dose.

## 3 RESULTS

### 3.1 Antimicrobial resistance in *Streptococcus* and detection of antimicrobial resistance genes

*Streptococcus* were tested for their susceptibility to seven antimicrobial agents before and after induction, and the results are shown in Table 3. All inducted strains were resistant to the antibiotics tested. The induced strains that demonstrated resistance to quinolones, the results are shown in Table 4. *S. agalactiae* (CVCC1886) had contained resistance genes before induction. The resistance phenotypes of *S. dysgalactiae* (CVCC3701) all reached the level of resistance, whereas no quinolone resistance genes were detected after induction. For the strains demonstrating resistance to β‐lactams, the results are shown in Table 5. *Pbp1a* and *Pbp2b* were identified from CVCC1886 before and after induction. CVCC3701 did not carry three resistance genes before induction, whereas *Pbp1a* and *Pbp2b* genes were detected by penicillin induction. In the erythromycin‐inducted resistant strains, *ermB*, *ermA* and *mefA* genes were not identified.

**TABLE 3:** MIC of bacterial resistance before and after induction

Strain	MIC (μg/ml)						
	NOR	OFLX	CIP	PEN	AMX	CEF	ERY
CVCC1886 (*S. agalactiae*)							
Before	4	2	0.5	4	4	<0.25	<0.25
After	64	256	128	64	128	8	64
CVCC3701 (*S. dysgalactiae*)							
Before	2	1	<0.25	1	1	<0.25	<0.25
After	64	256	128	64	128	8	64

Abbreviations: AMX, amoxicillin; CEF, cefalotin; CIP, ciprofloxacin; ERY, erythromycin; MIC, minimum inhibitory concentration; NOR, norfloxacin; OFLX, ofloxacin; PEN, penicillin.

**TABLE 4:** Resistance genes of quinolones

Resistance genes	*S. agalactiae*				*S. dysgalactiae*			
	CVCC1886	NOR	OFLX	CIP	CVCC3701	NOR	OFLX	CIP
GyrA	+	+	+	+	−	−	−	−
GyrB	+	+	+	+	−	−	−	−

Abbreviations: −, negative; +, positive; CIP, ciprofloxacin; NOR, norfloxacin; OFLX, ofloxacin.

**TABLE 5:** Resistance genes of β‐ lactam antibiotics

	
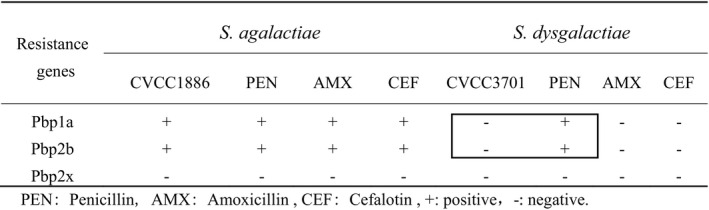

Abbreviations: −, negative; +, positive; AMX, amoxicillin; CEF, cefalotin; PEN, penicillin.

### 3.2 Detection of virulence genes

The strains before and after resistance induction were tested by PCR for the presence of seven genes potentially involved in virulence, and the results are presented in Table 6. Seven virulence genes were present in CVCC1886 and induced strains. In contrast, *cyl* gene was only identified in CVCC3701, all virulence genes were discovered in CVCC3701‐PEN and other induced strains did not change against CVCC3701.

**TABLE 6:** Virulence genes of strains before and after induced resistance

	
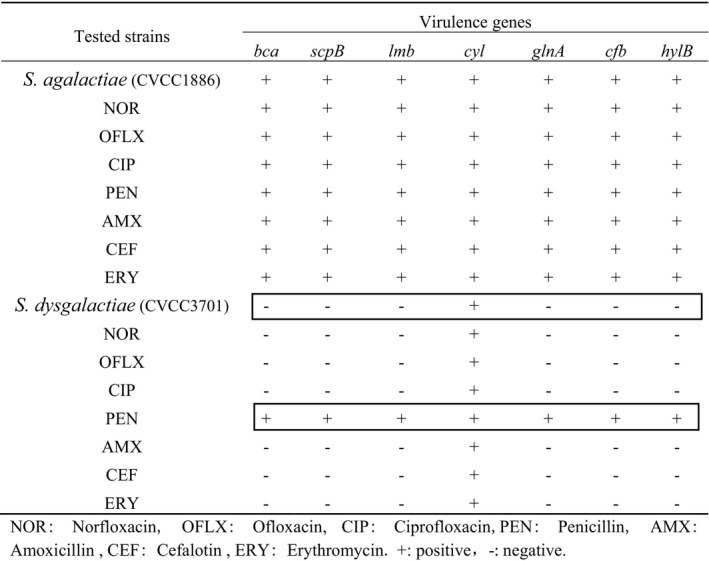

Abbreviations: −, negative; +, positive; AMX, amoxicillin; CEF, cefalotin; CIP, ciprofloxacin; ERY, erythromycin; NOR, norfloxacin; OFLX, ofloxacin; PEN, penicillin.

### 3.3 Detection of physiological and biochemical properties in strains before and after induction

According to Tables 5 and 6, CVCC3701 increased resistance genes *Pbp1a* and *Pbp2b*, virulence genes *bca, scpB, lmb, glnA, cfb, scaA* and *hylB* had been also found after being induced by penicillin. So CVCC3701 and CVCC3701‐PEN were selected as tested strains. Their appearance presented smooth, raised and round small colonies on the blood nutrient agar plate with typical rings of hemolysis. CVCC3701 and CVCC3701‐PEN were stained by the gram, and the bacteria were observed as round, blue‐purple, double or short chain arrangement under an oil microscope. The growth curve of tested strains was plotted by OD625 measurement, the growth and propagation rate of CVCC3701‐PEN was slower than that before induction.

### 3.4 Detection of LD50 on tested strains

The LD0 and LD100 of the tested strains were obtained by pretest. The LD0 and LD100 of CVCC3701 were 4.0 × 10^5^ and 1.0 × 10^8^ CFU/ml, whereas CVCC3701‐PEN were 1.4 × 10^8^ and 1.4 × 10^10^ CFU/ml, respectively. Bliss statistical software was used to analyse the data. The LD50 of tested strains are shown in Table 7. The results suggested that the LD50 of the induced strain was 1.1 × 10^2^ times higher than before, indicating that the virulence of the induced strain was lower than that before induction.

**TABLE 7:** Detection of LD50 in mice

Strains	Groups	Concentration (CFU/ml)	Number of mice	Inoculation method	Inoculation dose	Deaths number	LD_50_ (CFU/ml)
*S. dysgalactiae* CVCC3701	4‐LD_100_	1.0 × 10^9^	8	Intraperitoneal injection	0.5 ml	8	5.45 × 10^6^
	4‐A	1.4 × 10^8^	8			7	
	4‐B	1.98 × 10^7^	8			3	
	4‐C	2.8 × 10^6^	8			1	
	4‐LD_0_	4.0 × 10^5^	8			0	
*S. dysgalactiae* CVCC3701‐ PEN (penicillin)	5‐LD_100_	1.4 × 10^10^	8	Intraperitoneal injection	0.5 ml	8	5.82 × 10^8^
	5‐A	4.4 × 10^9^	8			7	
	5‐B	1.1 × 10^9^	8			6	
	5‐C	4.4 × 10^8^	8			3	
	5‐LD_0_	1.4 × 10^8^	8			0	

## 4 DISCUSSION

With the wide application of antimicrobial agents in clinical therapy and prevention, the antimicrobial resistance has been increasing, especially the unreasonable use of antibiotics. The emergence of resistance brings great obstacles to the clinical treatment of diseases, but also endangers the health of humans and animals (Ding et al., 2016; Liu et al., 2019). The World Health Organization (WHO) has listed the emergence of resistance as one of the biggest public health security problems of the 21st century (Alejandro et al., 2013).

*Streptococcus* are pathogens and related to various animal infectious diseases, thereby reducing the production performance (Jain et al., 2012). In recent years, the abuse of antibiotics in livestock production has led to an increase in the resistance of *Streptococcus*, which has brought difficulties to prevention and control the *Streptococcus* caused diseases (Ding et al., 2016). Previous studies have demonstrated that the acquisition of antimicrobial resistance has an important effect on the change in their virulence (Alejandro et al., 2013). It has also been reported that the adsorption, colonization, invasion and tissue damage of pathogen are always related to virulence factors (Brooks & Mias, 2018). In regards to the *Streptococcus*, Rieux et al. (2001) found that the virulence of *Streptococcus pneumoniae* was significantly reduced due to the changes of *PbpX* and *Pbp2b* after the action of penicillin. Padilla et al. (2010) suggested that the efflux pump caused resistance while virulence would be increased. Meanwhile, Ismaeel et al. (2005) found that the ability of cytolethal distending toxin production by Campylobacter jejuni was enhanced when under the antibiotic sub‐inhibitory concentration of ofloxacin and erythromycin. The effect of sub‐inhibitory concentrations of cefalexin, ciprofloxacin and roxithromycin was investigated on coagulase, toxic shock syndrome toxin 1 (TSST‐1) expressed by *Staphylococcus aureus*, which may result in altered virulence states in pathogenic bacteria (Haddadin et al., 2010). However, there were limited reports about the effects of drug resistance and virulence in *S. agalactiae* and *S. dysgalactiae*.

In this study, the results showed that the resistance phenotype of *S. agalactiae* CVCC1886 changed from the previous sensitivity to resistance after induction. However, detection of resistance genes findings showed that it had been carried before induction. The reason might be due to the high sensitivity for the determination of the genetic level, whereas the determination of resistant phenotype was lower. In addition, our results indicated that the resistance phenotype of *S. dysgalactiae* CVCC3701 changed from the previous sensitivity to resistance after induction by amoxicillin, cefalotin, norfloxacin, ofloxacin, ciprofloxacin and erythromycin. Corresponding resistance genes were not detected before and after induction, which suggested that long‐term drug screening may be needed or resistance was mediated by other genes. Similarly, it has been reported that Sub‐MIC of antibiotics could affect the expression of resistance genes, such as penicillin acted on *Actinobacillus pleuropneumoniae*, which can up‐regulate the expression of *pgaA*, and affect the expression of *cpxR* and *cpxA* genes (Hathroubi et al., 2015). Also, the previous study suggested that *ErmC* gene expression was significantly up‐regulated by erythromycin on *Staphylococcus epidermidis* (He et al., 2014). Levofloxacin and ciprofloxacin acted on MRSA, the expression of *norR, mecA* and other genes would be up‐regulated (Tattevin et al., 2009). Quorum sensing is a process of cells communicate with each other that allows bacteria to share information about cell density and adjust gene expression accordingly. Sub‐inhibitory antibiotics activated the *lux* gene by the quorum induction promoter, regulated the transcription of bacterial genes and the expression of bacterial genes (Goh et al., 2002).

In this study, seven virulence genes were found in *S. agalactiae* CVCC1886, only *cyl* gene was detected in *S. dysgalactiae* CVCC3701 before and after induction, which indicated *S. agalactiae* might be more virulent. The resistance genes (*pbp1a* and *pbp2b*) were detected in the CVCC3701‐PEN, and the virulence genes were increased compared with those before induction. However, the virulence test showed that the virulence of the strain was reduced after induction, which was about 100 times. The virulence of *S. dysgalactiae* CVCC3701 was greatly weakened when resistance was induced in vitro. Similar to our study, it has been previously indicated that tetracycline and clindamycin significantly increased the expression of the *agr* virulence regulator at sub‐inhibitory concentrations, and increased the production of phenol‐soluble modulin cytolysins of MRSA (Joo et al., 2010). However, Ohlsen et al. (1998) revealed that β‐lactam up‐regulated alphatoxin (hla) gene expression of *Staphylococcus aureus*, while aminoglycoside down‐regulated *hla, lacZ* gene expression. Also, it was reported that low concentrations of antibiotics could also act as intraspecific or interspecific signalling molecule in bacteria (Rutherford & Bassler, 2012; Yim et al., 2007). These findings implied that the influence of sub‐inhibitory concentration of antibiotics on bacterial virulence could enhance or weaken, both of which are possible.

There might be several considerations: the expression of virulence factor decreased after induced resistance. Mutations in the genes of bacteria during induction might lead to abnormalities in the genes encoding the pathogenic factors or their regulators. For instance, Sakoulas et al. (2002) found that the decreased expression of virulence factor of vancomycin intermediate Staphylococcus aureus (VISA) was related to the abnormal function of the regulatory gene in *S. aureus*. Under the pressure of long‐term drug selection, bacteria are less virulent in order to adapt to the environment. In *Salmonella* typhimurium, the mutation of *rpsL* gene conferred resistance to streptomycin. The resulting amino acid substitution (K42N) in ribosomal protein S12 caused an increased rate of ribosomal proofreading, which slowed down protein synthesis, bacterial growth rate and virulence (Maisnier‐Patin et al., 2002). Some structures of bacteria (such as ribosomes, RNA polymerases, DNA rotating enzymes, cell walls, etc.) were changed when it develop resistance, resulting in reduced growth rate, colonization ability and pathogenicity of bacteria. In other words, the acquisition of resistance of bacteria requires the fitness cost (Enne et al., 2004). The decreased virulence of bacteria might be associated with the slower growth and reproduction rate. The *VanA* gene, which mediates vancomycin resistance in *S. aureus*, it can be divided into induced resistance and intrinsic resistance due to differences in operand level expression (Arthur et al., 1992). There were different types of *VanA* in MRSA, their adaptability is different, and the growth capacity of the bacterial strains decreased by 20%–38% after in vitro‐resistance induction. In contrast, the growth capacity decreased by only 0.04%–0.3% (Foucault et al., 2009). The growth and reproduction rate of VISA was significantly slower than that of the standard strain (Cui et al., 2003). Because of the slowing down of growth and reproduction, the amount of toxin released by bacteria is reduced, which is beneficial for the body to produce an immune response. Therefore, the relationship between bacterial resistance and virulence needs to be further studied.

## 5 CONCLUSIONS

To summarize, *S. dysgalactiae* (CVCC3701) is a susceptible strain of drug resistance to antibiotics, in vitro‐induced drug resistance reduced the virulence of CVCC3701, but the virulence is still exists and also could result in the death of mice. For the public health safety, it must be alert to the emergence of drug resistance of *Streptococcus* in animal production.

## CONFLICT OF INTEREST

The authors report that they have no conflicts of interest.

## AUTHOR CONTRIBUTION

**Yue‐Xia Ding:** Conceptualization; Data curation; Formal analysis; Methodology; Writing‐original draft; Writing‐review & editing. **Qun Wu:** Data curation; Methodology. **Yan Guo:** Writing‐review & editing. **Man Li:** Formal analysis. **Pei‐Feng Li:** Formal analysis. **Yi Ma:** Conceptualization; Funding acquisition; Supervision; Writing‐review & editing. **Wen‐Chao Liu:** Writing‐original draft; Writing‐review & editing.

## Funding information

This research was supported by 2019 Guangdong University Features Innovation Project by the Department of Education in Guangdong Province, China (2019KTSCX057) and the Project of Innovation and Strengthening of Guangdong Ocean University (Q18290).

### Peer Review

The peer review history for this article is available at https://publons.com/publon/10.1002/vms3.404.

## Data Availability

All public data generated or analysed during this study are included in this article. Data sharing is not applicable to this article as no new data were created or analysed in this study.
